# Supplementary material for: Genetic Characterization of the Partial Disease Resistance of Rice to Bacterial Panicle Blight and Sheath Blight by Combined QTL Linkage and QTL-seq Analyses
Source: Plants (Basel). 2023 Jan 26;12(3):559. doi: 10.3390/plants12030559 (PMC9920235; doi:10.3390/plants12030559)
Supplement: Supplementary file 1 [file plants-12-00559-s001.zip › plants-2050640-supplementary.pdf]

Table S1. Correlation of plant height (HT) to bacterial panicle blight (BPB) and sheath blight (SB) diseases from 2012 to 2014

	HT2012	HT2013	HT2014
**SB2012**	-0.21***	-0.01ns	0.02ns
**SB2014**	-0.16**	-0.13*	-0.15**
**BPB2012**	-0.18***	-0.14**	-0.14*
**BPB2013**	-0.14*	-0.10ns	-0.12*
**HT2012**	1	0.47***	0.39***
**HT2013**	0.47***	1	0.65***
**HT2014**	0.39***	0.65***	1

ns: not significant
***: significant at p-value= 0.001
**: significant at p-value= 0.01
*: significant at p-value= 0.05.

Table S2. Whole genome mapping statistics for the parents and selected RILs for 'Resistant' and 'Susceptible' bulks

Sample	SB mean	BPB mean	HEAD mean	Bulk	Total reads	Mapped reads	Mapping ratio (%)	Properly paired (%)	Average coverage (x)
**TJ-RIL3**	4.25	3.92	97.5	Resistant	33,713,140.00	32,630,542.00	96.79	94.71	49.53
**TJ-RIL38**	3.75	3.67	102.25	Resistant	28,601,487.00	28,557,186.00	54.9	53.49	49.66
**TJ-RIL40**	3	3.92	92.75	Resistant	32,472,416.00	25,683,076.00	79.09	77.61	49.79
**TJ-RIL58**	2.75	3.92	103	Resistant	36,032,575.00	20,843,587.00	57.85	56.59	49.92
**TJ-RIL88**	2.25	4.17	102	Resistant	24,666,700.00	23,818,609.00	96.56	94.96	49.05
**TJ-RIL109**	3.5	4.17	101.5	Resistant	40,813,643.00	39,315,553.00	96.33	94.51	48.18
**TJ-RIL173**	2.5	2	106.5	Resistant	32,540,849.00	12,622,981.00	38.79	37.96	48.31
**TJ-RIL239**	4	3.17	101	Resistant	26,851,705.00	26,452,733.00	98.51	96.79	48.44
**TJ-RIL80**	7.75	7.42	95.25	Susceptible	35,100,111.00	28,320,943.00	80.69	78.44	48.57
**TJ-RIL125**	7.5	5.42	94.75	Susceptible	37,265,217.00	34,278,098.00	91.98	90.3	49.7
**TJ-RIL202**	9	7.58	91.75	Susceptible	33,332,943.00	30,632,186.00	91.9	89.9	48.83
**TJ-RIL241**	8.75	5.33	96.25	Susceptible	37,429,310.00	35,915,445.00	95.96	94.16	48.96
**TJ-RIL255**	8	5.33	95	Susceptible	30,187,592.00	12,609,030.00	41.77	40.91	49.09
**TJ-RIL288**	7.75	5.42	95.5	Susceptible	27,883,400.00	27,341,831.00	98.06	96.2	49.22
**TJ-RIL289**	9	6.5	93.5	Susceptible	25,624,237.00	24,726,694.00	96.5	94.85	49.35
**Resistant Bulk Total**					255,692,515.00	197,068,450.00	77.07	75.53	49.48
**Susceptible Bulk Total**					226,822,810.00	193,824,227.00	85.45	83.7	49.61
**Jupiter**	2.29	2.39	101.5	Resistant	78,995,513.00	78,122,496.00	98.89	97.33	20.09
**Trenasse**	8.46	7.6	96.5	Suscpetible	84,628,145.00	83,314,224.00	98.45	96.05	21.43

Table S3. T-test for phenotypic differences between resistant and susceptible bulks

**Trait**	**Mean of Resistant Bulk / Mean of Susceptible Bulk**	***P* value**
**Bacterial Panicle Bight**	3.25 / 8.25***	1.5376E-09
**Sheath Blight**	3.57 / 6.14***	0.000106874
**Days to 50% heading**	100.81 / 94.57***	0.001490314

*** Significant at $P < 0.001$.

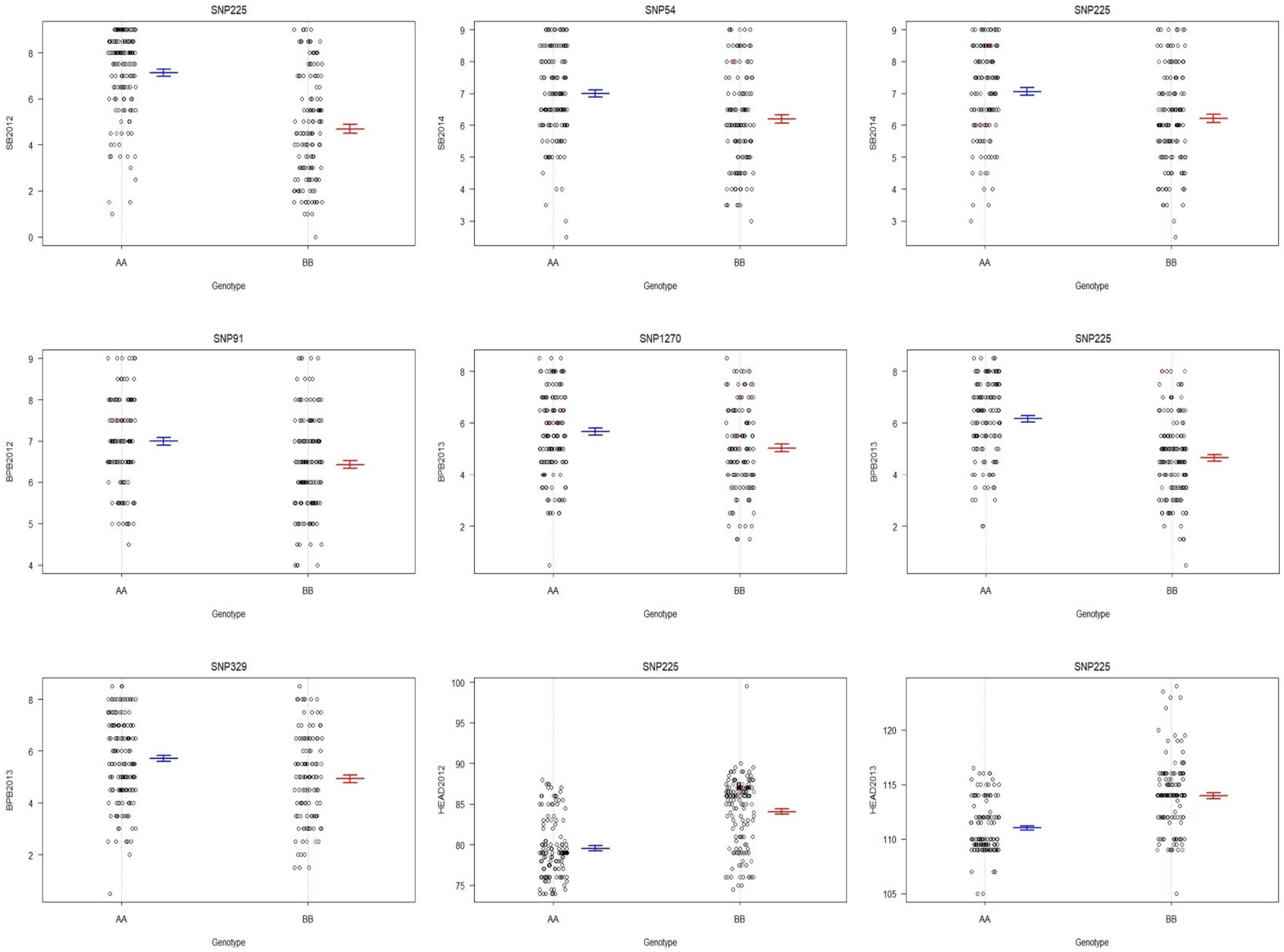

Figure S1. The effect plot of markers associated with the significant positions for bacterial panicle blight, sheath blight and days-to-heading identified by non-parametric analysis (Kruskal-Wallis test). The AA and BB indicate Trenasse and Jupiter genotypes, respectively.

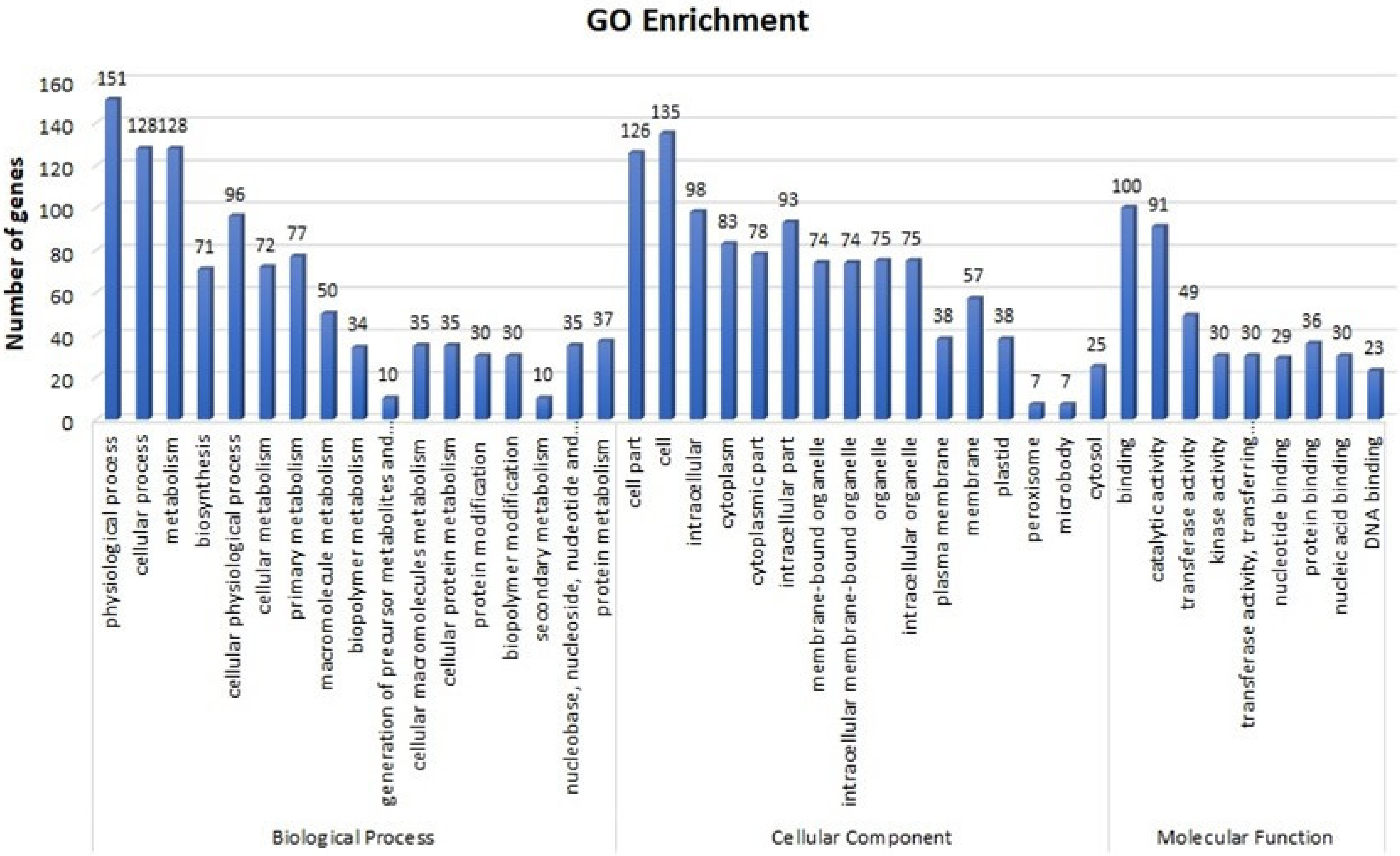

Figure S2. Single enrichment analysis for biological process, molecular functions and cellular components of the major QTL interval found in chromosome 3 for bacterial panicle blight, sheath blight and day-to-heading from the linkage mapping of the Trenasse/Jupiter RIL population.

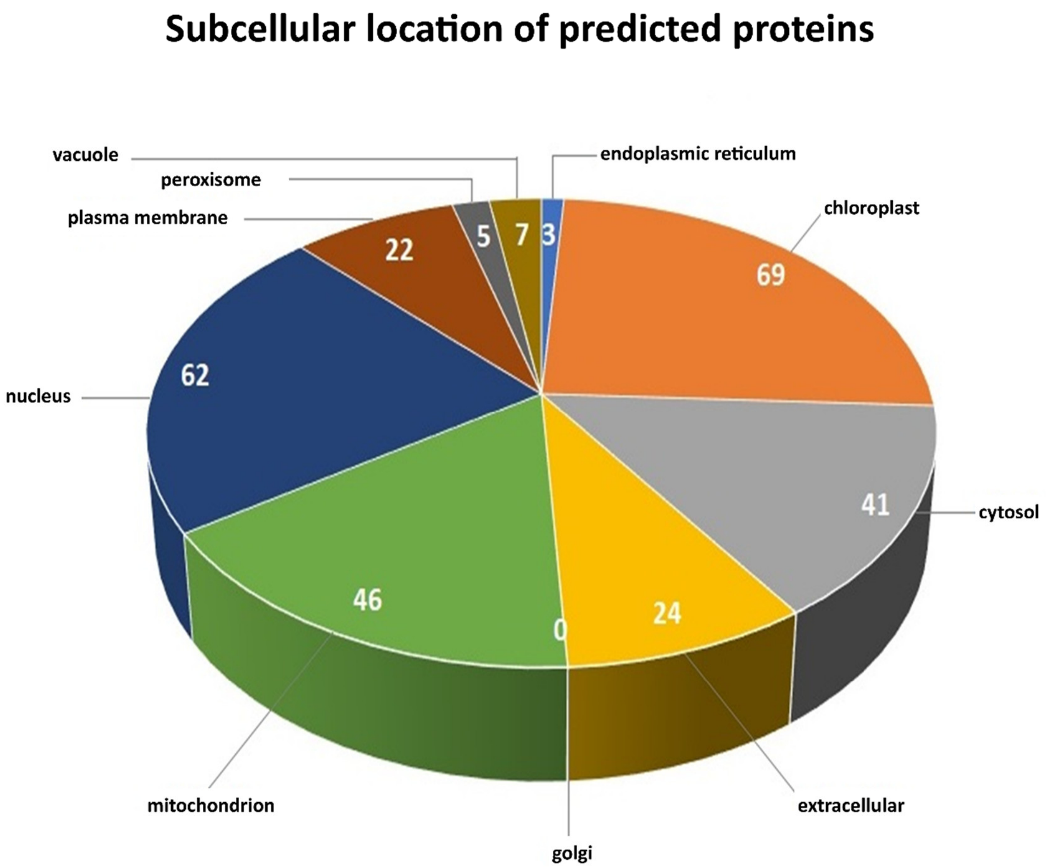

Figure S3. The predicted subcellular locations of the 201 proteins identified in the major QTL on chromosome 3.
